# The impact of periodontitis on vascular endothelial dysfunction

**DOI:** 10.3389/fcimb.2022.998313

**Published:** 2022-09-02

**Authors:** Qian Li, Xiangying Ouyang, Jiang Lin

**Affiliations:** ^1^ Department of Stomatology, Beijing Tongren Hospital, Capital Medical University, Beijing, China; ^2^ Department of Periodontology, Peking University School and Hospital of Stomatology, Beijing, China

**Keywords:** periodontitis, *Porphyromonas gingivalis*, vascular endothelial cells, vascular endothelial dysfunction, vascular disease

## Abstract

Periodontitis, an oral inflammatory disease, originates from periodontal microbiota dysbiosis which is associated with the dysregulation of host immunoinflammatory response. This chronic infection is not only harmful to oral health but is also a risk factor for the onset and progress of various vascular diseases, such as hypertension, atherosclerosis, and coronary arterial disease. Vascular endothelial dysfunction is the initial key pathological feature of vascular diseases. Clarifying the association between periodontitis and vascular endothelial dysfunction is undoubtedly a key breakthrough for understanding the potential relationship between periodontitis and vascular diseases. However, there is currently a lack of an updated review of their relationship. Therefore, we aim to focus on the implications of periodontitis in vascular endothelial dysfunction in this review.

## Introduction

Periodontitis is a common inflammatory oral disease associated with periodontal microbiota dysbiosis and host immune response dysregulation (Papapanou et al., 2018). Periodontal dysbiosis signifies the shift from a symbiotic to a dysbiotic microbial community, resulting in the transition from a periodontal healthy state to inflammation (Slazhneva et al., 2020). Moreover, host immunological and genetic mechanisms have been further discerned as contributory factors for periodontitis (Sedghi et al., 2021b). Periodontal infection is not only harmful to oral health but is also linked to a number of systemic diseases. Periodontal medicine, a term defined to discover how periodontal infection affects extraoral health, is therefore considered (Monsarrat et al., 2016; Beck et al., 2019). Over the past decades, great progress has been made in periodontal medicine. Up to date, over 50 systemic diseases are now being researched regarding their relation to periodontal diseases (Loos, 2016; Fi and Wo, 2021).

The role of periodontal inflammation in vascular pathology has been consistently highlighted. Vascular endothelial cells (VECs), a layer of cells lining the lumen of the blood vessel, play an important role in vascular diseases. They have active metabolism and can secrete various factors to regulate cell migration and adhesion, thrombosis, smooth muscle cell proliferation and migration, and vascular wall inflammation, which are extremely significant for vascular homeostasis (Krüger-Genge et al., 2019; Hennigs et al., 2021). When responding to adverse stimuli, the phenotype of VECs changes to an activated one, i.e., endothelial dysfunction (ED) (Corban et al., 2019; Medina-Leyte et al., 2021). ED has been demonstrated to have initiating and promoting effects on the occurrence and development of vascular diseases, such as atherosclerotic disease, hypercholesterolemia, diabetes, and hypertension (Lyle and Taylor, 2019).

In addition to traditional risk factors, inflammatory diseases, including periodontitis, are closely related to ED development (Paul et al., 2020). A large number of epidemiological studies and clinical evidences have confirmed the correlation between periodontitis and ED. However, a causative link between periodontal infection and endothelial cells and the direct molecular mechanism of periodontitis role in ED remain unclear. This review thus summarizes the possibility of the link between periodontitis and ED.

## Periodontitis

Periodontitis is a very common biofilm-associated infection of the periodontium. It is now the sixth most prevalent disease globally, affecting about 50% of the world population (Balta et al., 2021). Despite its high prevalence, periodontitis is not taken seriously enough in the early stage, and most patients seeking treatment are in advanced stages of the disease. Approximately 11% of the world population suffer from severe periodontitis, which is the main reason leading to tooth loss and life quality reduction (Sanz et al., 2020). Periodontitis is characterized by inflammatory destruction of the tooth-supporting tissues, and its clinical features comprise gingival bleeding, periodontal pocket formation, clinical attachment loss, alveolar bone absorption, and even tooth mobility and loss (Slots, 2017; Kwon et al., 2021).

Periodontitis is a microbial-shift disease owing to polymicrobial dysbiosis (Lamont et al., 2018; Sedghi et al., 2021a). During the transition from periodontal homeostasis to dysbiosis, although the affected sites have greater microbial diversity and richness or present no significant difference, these sites exhibit unique microbial community structural characteristics. Specific genera, including *Porphyromonas, Treponema*, *Campylobacter*, *Eubacterium*, and *Tannerella*, have been identified at high levels in periodontitis sites, while other genera, such as *Veillonella, Neisseria, Rothia, Corynebacterium*, and *Actinomyces*, were highly prevalent in the healthy gingival sulcus (Plachokova et al., 2021; Abusleme et al., 2021). The characteristic of the periodontal microbiota is thus an ideal predictor of periodontal status. Nevertheless, the underlying mechanisms keeping the stability of and triggering the change in the microbial community are still not well understood. The inhibitory phenotype of *P. gingivalis*, *Tannerella forsythia* (*T. forsythia*), and *Treponema denticola* (*T. denticola*), namely the red-complex periopathogens, against the host innate response might play a pivotal role during the transition from periodontal health to disease (Xu et al., 2020; Prucsi et al., 2021). Moreover, community-based attack of periodontal pathogens on the host also offers a new possibility for periodontal microbial shift. The inoculation of *Porphyromonas gingivalis* (*P. gingivalis*, a keystone periodontal pathogen) with *T. denticola* (Verma et al., 2010) or *S. gordonii* (Lamont et al., 2010) has led to enhanced periodontal inflammation compared with *P. gingivalis* alone. Further research is still needed to elaborate on the biological mechanism of the dynamic change in the periodontal microorganisms.

More importantly, periodontitis presents a systematic chronic low-grade infection burden. Evidence-based literature has identified that periodontitis is not only a common oral health problem but also a risk factor implicated in multiple systemic cardiovascular diseases, such as hypertension, diabetes, and stroke (Thomas et al., 2015; Priyamvara et al., 2020; Del Pinto et al., 2020). Cardiovascular diseases are the biggest killers of human life and health worldwide, and they also remain the major public health problems in both developed and developing countries (Roth et al., 2020). The risk for cardiovascular disease is increased in periodontitis patients (Gheorghita et al., 2019; Sanz et al., 2020).

## Vascular endothelial dysfunction

VECs lining the inner layer of blood vessels are the main regulator of vascular and organ homeostasis. The investigation of the implications of periodontitis in vascular endothelial function is undoubtedly a key breakthrough for the potential relationship between periodontitis and cardiovascular diseases.

The endothelium is in direct contact with blood flow and forms a barrier between blood and underlying tissues. Under quiescent conditions, VECs sense and transduce signals between blood and tissues, regulate the trafficking of cells in blood, and maintain a non-thrombogenic blood vessel surface (Zhu and Lee, 2016; Hennigs et al., 2021). When perturbed, these cells respond rapidly to various stimuli, such as microbial components, cytokines, oxidized low-density lipoproteins, immune complexes, and mechanical damage, to maintain vascular homeostasis (Eelen et al., 2018; Shao et al., 2020). However, exaggerated response of VECs may finally result in ED. The inflammatory reaction is the main characteristic of vascular ED. Vascular inflammation involves the onset of signaling cascades triggered by endothelial signaling, leading to increased production of cytokines, chemokines, and cell adhesion molecules, finally directing the recruitment of inflammatory cells (Eelen et al., 2018). Additionally, this process is also accompanied by the up-regulation of reactive oxygen species, endothelin, lipid peroxidation, and thrombus regulatory protein and the impaired production of nitric oxide (NO) (Bondareva and Sheikh, 2020).

VEC dysfunction provides favorable conditions for increased endothelial permeability, augmented immune cell adhesion, platelet activation, activation of coagulation and fibrinolytic systems, lipid deposition, vascular vasomotor disorder, proliferation and migration of smooth muscle cells, and deposition of extracellular matrix, finally resulting in vascular diseases, such as hypertension, atherosclerosis, and coronary arterial disease (Favarato, 2018; Corban et al., 2019; Krüger-Genge et al., 2019). The pathologic state of dysfunctional endothelium as an early pathologic change occurring before detectable morphologic changes in the blood vessel wall is thought to be an independent predictor of the risk and prognosis of cardiovascular diseases. For example, ED has been observed in patients with hypertension, dyslipidemia, diabetes mellitus, and inflammatory diseases (Haas et al., 2018). And abnormal vascular endothelial function is a known prognostic indicator in children with familial cardiomyopathies (Tavares et al., 2012). Additionally, ED can also be used to predict future restenosis and major cardiovascular events in acute coronary syndrome patients treated with percutaneous coronary intervention (Yamamoto et al., 2014; Cheng et al., 2018).

Clinical assessment of endothelial function is an important insight into the patient’s vascular status. The most widely applied indicators for endothelial function measurement are flow-mediated dilation (FMD) and nitroglycerin-mediated dilation (NMD) of the brachial artery. Both of them are performed by measuring macrovascular endothelial function with brachial artery ultrasound (Ambrosino et al., 2021). In contrast, the application of reactive hyperemia-peripheral arterial tonometry (RH-PAT), evaluating the ratio of blood flow volume of microvascular endothelium before and after blood flow release, offers a simpler assessment approach. However, there is currently no clinical guideline-based recommendation for vascular endothelial function testing, and more work are required to develop such a guideline. In addition to clinical assessment, many laboratory biomarkers can also be applied to ED evaluation. These biomarkers contain vascular cell adhesion molecule-1 (VCAM-1), intercellular cell adhesion molecule-1 (ICAM-1), pentraxin-3, e-selectin, von Willebrand factor-1 (vWF), asymmetrical dimethylarginine (ADMA), angiopoietin-1 (Ang-1), thrombomodulin, endothelial microparticles (EMPs), and endothelial progenitor cells (Balta, 2021). Noteworthily, there are two novel biomarkers: endocan and endoglin. Endoglin is a transmembrane receptor for transforming growth factor β-1 and 3 in VECs (Jeng et al., 2021). The long-form endoglin (L-endoglin) and short-form endoglin (S-endoglin) are two isoforms of endoglin. L-endoglin is undetectable in resting ECs but is highly expressed in ECs at sites of angiogenesis, upon inflammation or ischemic stimuli (Ollauri-Ibáñez et al., 2017). Endocan is a soluble proteoglycan secreted by vascular ECs (Çimen et al., 2016). The expression of endocan in ECs can be upregulated in response to inflammatory triggers, such as lipopolysaccharide and cytokines (Meurer and Weiskirchen, 2020). Both of endoglin and endocan are suggested as possible biomarkers for ED (Leite et al., 2020). However, they are not VECs-specific and can be expressed in other cells like monocytes and bronchi epithelial cells respectively, so more research is necessary to evaluate their predictive value and reproducibility in vascular diseases. In a word, all of the current potential indicators have not been proven to be a causal risk factor for cardiovascular disease, although they are highly associated with worsening vascular endothelial function.

## The implication of periodontitis in vascular endothelial function

Epidemiological studies and clinical cohort and case-control evidences have suggested that periodontal treatment can be an effective measure for ED improvement. Additionally, the biological plausibility of periodontitis impact on VECs has been gradually revealed.

### The effect of periodontal therapy on vascular endothelial function

Epidemiologic evidences support that people with periodontitis have a higher prevalence of subclinical cardiovascular disease, peripheral artery disease, and coronary events (Sanz et al., 2020). In a large longitudinal population-based study, periodontitis has been significantly associated with high FMD levels (Holtfreter et al., 2013). Moreover, in an update pilot study, increased tooth mobility has been independently correlated with endothelial dysfunction using RH-PAT after adjustment for age and glycosylated hemoglobin (HbA1c) (Fujitani et al., 2020). Ronaldo et al. (Lira-Junior et al., 2014) have evaluated endothelial function in severe chronic periodontitis patients. They have found that severe periodontitis was associated with nailfold and gingival microvascular and endothelial dysfunction. Specifically, there was a decrease in functional capillary density, capillary diameters, red blood cell velocity at rest, endothelium-independent vasodilatation, and post-ischemic peak flow in patients with periodontitis. Other experimental findings have also supported the passive impact of periodontitis on function of vascular endothelium (Brito et al., 2013; Lira-Junior et al., 2014; Parvaneh et al., 2021).

Intensive periodontitis treatment, consisting of oral hygiene education, scaling, and root planing, has been suggested to improve endothelial function by the vast majority of trials. Potential biomarkers linking periodontitis with endothelial dysfunction, including C-reactive protein (CRP), interleukin (IL)-1, ICAM-1, E-selectin, vWF, plasminogen activator inhibitor type-1 (PAT-1), and plasminogen, have been found to be decreased after periodontal treatment (Tonetti et al., 2007; Li et al., 2011b; D'Aiuto et al., 2013; Hansen and Holmstrup, 2022). More than 10 years ago, Tonetti et al. (2007) showed that improved endothelial function paralleled periodontal health 60 and 120 days after periodontal therapy, although ED and an increase in inflammatory factors were observed 24 h after periodontal treatment. The immediate ED might be caused by the acute, transient systemic inflammation after periodontal treatment, and the improved endothelial function ultimately benefits from good oral health after 2 months of therapy. Additionally, the number of missing teeth, an easily accessible clinic marker, has been reported to be correlated with higher coronary artery calcium score (CACS) (Donders et al., 2020). Combining the result of this explorative pilot study with additional clinical information and biomarkers might contribute to the further exploration of the relationship between missing teeth and ED. The result of Matsui et al.’s study is appealing as well. Their result has shown that low frequency and short duration of tooth brushing were associated with an increased odds ratio of a low FMD after conventional risk factors adjustment (Matsui et al., 2017). This conclusion was in line with the findings of Kajikawa (Kajikawa et al., 2014). Their research achievement has well confirmed the passive impact of poor oral health on ED, but more large-scale clinical studies are needed. Furthermore, several clinical trials have confirmed the positive effect of periodontal treatment on endothelial function in groups of periodontitis with other co-morbidities. Endothelial function improvement and inflammatory biomarkers reduction have been observed after periodontal treatment of subjects with both periodontitis and cardiovascular diseases, and this improvement sustained well over half a year after therapy (Teeuw et al., 2014). In addition, EMPs, together with systolic and diastolic blood pressure (BP), have also been found to be markedly reduced by subgingival scaling and root planing (without antihypertensive medication therapy) in prehypertensive patients with periodontitis, and the reduction in EMPs and BP levels has been significantly related to the improvement in pocket depth (Zhou et al., 2017).

There are surely contradictory findings. A study has reported that no significant improvement in vascular endothelial function could be confirmed after periodontal treatment in patients with moderate-to-severe periodontitis (Li et al., 2011a). We suggest two possible reasons for their different results. First, their assessment criterion of periodontitis degree (half-mouth method at three sites per tooth) has been different from that in other studies (full-mouth periodontal recordings), which might be the most potential influence factor. The difference might also be explained by the study population, which has been compromised by the broad age range and even confounding factors, such as smoking, cardiovascular risk factors, diabetes mellitus, and chronic kidney disease. In another 3-month follow-up period, it has also not been shown that nonsurgical periodontal therapy improved FMD in patients with coronary disease (control 1.37% *vs.* test 1.39%) (Saffi et al., 2018). In this study, the selected individuals were suffering from periodontitis and chronic heart disease and even have already been receiving cardiovascular treatments. Regular cardiovascular therapies may explain the absence of significant between-group differences.

Generally, most existing clinical trials have tended to include participants affected by chronic severe generalized periodontitis. However, whether the improvement degree of vascular function by periodontal therapy is influenced by periodontitis severity remains unknown. Furthermore, current understanding of the effect of periodontal therapy on ED is mainly based on patient comparative and treatment clinical studies. More large-scaled and well-designed cohort studies and clinical trials with improved design in multicenter groups are indispensable.

### The influence of periodontitis on vascular endothelial function

Periodontitis can contribute to or increase endothelial inflammation. Periodontal pathogens and their noxious stimuli or periodontal cytokines can be detected by receptors on vascular endothelial cells, leading to the activation of an inflammatory cascades. The most well-characterized specialized pattern-recognition receptors (PRRs) are toll-like receptor-2 (TLR-2) and TLR-4, which play a key role in periodontal bacterial recognition (Hajishengallis et al., 2006; Hajishengallis and Lambris, 2011; Chen et al., 2021). Nucleotide-binding leucine-rich repeat receptors (NLRs) and scavenger receptors (SRs) are also involved in ED induced by periodontal infection (Zelkha et al., 2010; Huck et al., 2015; Li et al., 2020). After the recognition of noxious substances from the periodontium, the release of an inflammatory cytokine network is initiated, which can result in a complex proinflammatory and prothrombotic phenotype of endothelial cells. For example, tumor necrosis factor (TNF)-α, IL-1, IL-6, and IL-8 released by periodontal bacteria can invade the endothelial layer (Chhibber-Goel et al., 2016) and promote the expression of chemokines and adhesion molecules, including ICAM-1, VCAM-1, lymphocyte function-associated antigen 1 (LFA-1), P-selectin, and E-selectin (Schenkein and Loos, 2013). These chemokines and adhesion molecules can be induced or increased by lipopolysaccharide (LPS) and endothelial microvesicles (MVs) of *P. gingivalis* as well (An et al., 2014; Bugueno et al., 2020). Moreover, *P. gingivalis* infection can also modulate the production of inflammatory cytokines, such as IL-1, IL-6, TNF-α, myeloperoxidase, and matrix metalloproteinase 2 (MMP-2)/tissue inhibitor of metalloproteinases 2 (TIMP-2) complex, and chemokines, such as monocyte chemotactic protein-1 (MCP-1), IL-8, and CX3C chemokine ligand 1 (CX3CL1), in VECs (Hashizume et al., 2011; Moura et al., 2017; Pan and Yan, 2019). The release of inflammatory factors further induces the migration and adhesion of leukocytes and monocytes to the intimal layer of the blood vessel. These immune cells can transport periodontal bacteria into the lesion and secrete more inflammatory factors at the same time, ultimately exacerbating endothelial inflammation. Moreover, ICAM-1 can bind to fibrinogen and reduce the expression of actin-associated endothelial tight junction proteins, such as occludin and zonula occludens-1, to increase endothelial layer permeability (Patibandla et al., 2010; Leite et al., 2020). In an *in vitro* model, LPS has also been found to induced caspase-mediated cleavage of adherens junction proteins (Ding et al., 2020). Other studies have ever reported that the gingipains and outer membrane vesicles of *P. gingivalis* mediated increased vascular permeability *via* a mechanism that involves proteolytic cleavage of the platelet endothelial cell adhesion molecule 1 (PECAM-1) (Yun et al., 2005; Farrugia et al., 2020; Zhang et al., 2021)

The increased permeability of the endothelium creates conditions for the coagulation and fibrinolytic systems activation, smooth muscle cells (SMCs) migration into the intima, lipoprotein flux, and foam cell formation. The coagulation and fibrinolytic system includes fibrinogen, vWF, tissue plasminogen activator (tPA), PAI-1, and coagulation factors VII and VIII. They play a vital role in maintaining vascular homeostasis. PAI-1 is one of the best-established fibrinolytic members and risk factors for vascular diseases. *P. gingivalis* infection can significantly reduce PAI-1 levels in human endothelial cells, and the degradation of PAI-1 will induce permeabilization and dysfunction of the vascular endothelial cells *via* the low-density lipoprotein receptor-related protein (Song et al., 2021). Fibrinogen is another important member of the coagulation and fibrinolytic system. Elevated fibrinogen is followed by an increased blood viscosity and shear stress, which, in turn, activate endothelial cells and platelets (Paraskevas et al., 2008; Luyendyk et al., 2018). Periodontitis has been reported to present with higher plasma fibrinogen levels and white blood cell counts than controls (Jayaraman et al., 2021). The increased fibrinogen can further stimulate the production of MCP-1, IL-6, IL-8, TNF-α, MMP-1, and MMP-9 (Patibandla et al., 2010; Luyendyk et al., 2018; Surma and Banach, 2021), and aggravate endothelial inflammation. A vicious pathogenic cycle is thus formed where the damaged coagulation and fibrinolytic system and endothelial inflammation reinforce each other by the positive feedback loop between them.

Periodontitis has also been strongly associated with an increase in the endothelial synthesis of Reactive oxidative stress (ROS) and the reduction in NO bioavailability. ROS is an important factor in causing ED. Excessive ROS accumulation interferes with the nitric oxide (NO) signaling pathway, thereby reducing NO bioavailability and leading to ED and endothelium-dependent relaxation reduction (Garcia and Sessa, 2019). As described by Xie et al. (Xie et al., 2020), increased mitochondrial ROS production has been observed in endothelial cells infected with *P. gingivalis*. Furthermore, salivary NO concentration has been reported as a potential linkage between periodontitis and ED (Moura et al., 2017). The reduced bioavailability of NO can inhibit the expression of adhesion molecules and promote SMCs migration and proliferation (Liccardo et al., 2019; Yang et al., 2022; Suh et al, 2019), further aggravating aberrant function of VECs. Recently, Parvaneh et al. (Parvaneh et al., 2021) established periodontitis in 8-week-old ApoE^-/-^ mice and showed that periodontitis exhibited impaired endothelial-dependent vasorelaxation responses to acetylcholine, which was indicative of NO bioactivity impairment and the onset of ED. Similarly, Campi et al. (Campi et al., 2016) have found that after 7 days of the induction of periodontitis, the vascular response of adult rat aorta was impaired in terms of norepinephrine-induced contraction and acetylcholine-dependent relaxation, and the endothelium-derived NO and cyclooxygenase 2 (COX-2) were involved in the process (Zhou et al., 2019; De Oliveira et al., 2021). Nuclear factor erythroid-derived 2-like 2 (Nrf-2) is a key transcriptional factor protecting cells from oxidative stress and influencing vascular endothelium homeostasis (Kovac et al., 2014). Periodontal infection can lead to impaired vascular relaxation *via* the glycogen synthase kinase 3β (GSK-3β)/tetrahydrobiopterin (BH4)/nitric oxide synthase (eNOS)/Nrf2/NOS pathways (Kovac et al., 2014), which may contribute to a potential new therapeutic strategy for periodontitis-induced ED.

Interestingly, Pereira et al. (Pereira et al., 2011) discovered that no significant changes in endothelium-dependent vasodilation were observed in 18-week-old mice with *P. gingivalis* over 12 weeks. The authors have suggested that the opposing results different from other studies might originate from the older mice used. In 18-week old adult mice, the senescence might begin, and even significant vascular pathology might have already been established (Pereira et al., 2011). However, significant ED has been observed in periodontitis-treated middle-aged (57-week) rats, in which increased nicotinamide adenine dinucleotide phosphate oxidase (NADPH oxidase) and COXs, downregulated eNOS and NO, and endothelium-derived hyperpolarizing factor-mediated vascular relaxation were found (Silva et al., 2021). Interestingly, Brito et al., (2013) have revealed that some systemic inflammatory markers and oxidative stress products returned to basal levels at day 28 after periodontitis establishment in a 10-week rat. This might be a consequence of host resistance to periodontal infection and inflammatory stimuli. Moreover, the activation of the endothelial calcium-activated potassium channel might be the key mediator for the recovery of VEC impairment (Jr et al., 2018). These findings together make the result of Pereira more comprehensible. And there is also a thought-provoking question that whether there are limitations to applying endothelial-dependent relaxation as a marker of endothelial dysfunction.

### The mechanism of periodontitis affecting vascular endothelial dysfunction

The biological pathways by which periodontitis accelerates vascular diseases have not been fully elucidated so far. To date, there are three plausible hypotheses, including the bacteriological, inflammatory, and immunological theories (Hajishengallis, 2015; Febbraio et al., 2022).

The bacteriological hypothesis postulates that the entry of periodontal pathogens into the bloodstream activates endothelial inflammatory response by multiple mechanisms, resulting in ED. The inflammatory theory favors that inflammatory mediators in infected periodontium are released into the systemic circulation, in turn, affecting endothelial function. The ulcerated periodontal pocket epithelium is one of the main accesses whereby periodontal pathogens, noxious products, and inflammatory cytokines enter the blood circulation. The ulcerated periodontal pocket epithelial area in patients with severe periodontitis is about 18–28 cm^2^ (Leira et al., 2018). This niche harbors 1×10^8^–1×10^10^ bacteria feeding on the inflammatory spoils (Hajishengallis, 2015). Periodontal microorganisms and their noxious products can enter the blood circulation from the ulcerated area during chewing, brushing, or invasive dental therapy (Dyke and Winkelhoff, 2013). Then, they cause chronically sustained systemic infection. This persistent low-level inflammation has detrimental effects on the blood vessel endothelium. However, it is challenging to discriminate the role of bacteria from the inflammatory response in ED. The specific pharmacotherapeutic interventions might shed light on this troublesome matter.

The detection of periodontal pathogens in atheromatous plaques from patients further supports the hypothesis above (Padilla et al., 2010; Rao et al., 2021). Moreover, these pathogens also have been shown to invade and survive in endothelial cells *in vitro* (Velsko et al., 2014; Dorn et al., 1999; Schenkein et al., 2020). Results from mice with periodontitis have further confirmed the systemic dissemination of periodontal bacteria within aortic endothelial cells (Chukkapalli et al., 2014; Velsko et al., 2014; Velsko et al., 2015). The question of how periodontal bacteria exit from ulcerated periodontal epithelium to systemic circulation is currently broadly emphasized and explored. Current evidences have suggested that periodontal bacteria can exploit recirculating monocytes (Suwatanapongched et al., 2010), erythrocytes (Ganuelas et al., 2013), and dendritic cells (Carrion et al., 2012) for dissemination. These cells engulf bacteria and transport them to distal vascular endothelial cells. Fimbriae protein possessing adherence and invasive properties may play a key role in this process, as the fimA-deficient mutant strain of *P. gingivalis* failed to adhere and invade cells (Jotwani and Culter, 2004; Hasegawa and Nagano, 2021). The transmission of *P. gingivalis* among cells can be mediated by membranous projections (Yilmaz et al., 2006) and autophagosomes (Takeuchi et al., 2011). More recently, research has shown that *P. gingivalis* was first encapsulated by early endosomes immediately upon their entry into cells, and then some of them were sorted to late endosomes for degradation, whereas others escaped from cells for further dissemination (Takeuchi et al., 2016). This discovery further reveals the recycling pathways by which intracellular bacteria exit infected cells. With such a dynamic, *P. gingivalis* can control its population in infected cells and allow for persistent infection.

The immunological hypothesis is based on the fact that the host immune response in susceptible individuals favors vascular inflammation. Although microbial plaque is indispensable in vascular pathology related to periodontitis, it is the host immune system that primarily drives the outcome of microbial infection. This is the underlying reason why patients are not equally susceptible and do not respond similarly to the same treatment (Dyke, 2020). There is a phenotype of hyper-inflammatory monocytes when the host is challenged by the periodontal bacteria, which can result in an abnormal release of a high amount of proinflammatory mediators (Jagannathan et al., 2014). People with this phenotype of monocytes have a higher risk of suffering from periodontitis and ED (Gurav, 2014). Noz et al. deepen this theory. They applied trained immunity to describe that monocytes/macrophages build immunological memory after encountering a pathogen, resulting in a persistent hyper-responsive phenotype (Noz et al., 2021). The results of them revealed that *P. gingivalis* can induce trained immunity in human monocytes, in terms of an augmented cytokine production capacity (Noz et al., 2021). These augmented cytokine production can further promote Th1 responses to increase macrophages activation to enhance inflammation in the vessel (Tonetti and Dyke, 2013). And it is worth noting that antibodies produced by adaptive immune cells may be cross-reacting with endothelial cells to enhance inflammation at the same time. For example, the autoimmune reaction against the heat-shock proteins (HSPs) is responsible for periodontitis-related ED. HSPs can activate dendritic cells and natural killer cells and play a major role in MHC-antigen processing and presentation (Bolhassani and Agi, 2019). They are protective and function as chaperones under physiological conditions, and their expression would enhance in response to various physical, chemical and microbial stimuli (Finlayson-Trick et al., 2019). Bacterial HSP60 (GroEL) of periodontopathic bacteria is homologous with the host HSPs and displays a strong immunogenic nature. The homology between the host HSP60 expressed by the ECs and GroEL in periodontal microorganism is unrecognizable by the host T cells (Ford et al., 2010). Thus, the antibodies directed against the bacterial GroEL cross-reacts with HSP60 on ECs, finally resulting in autoimmune responses that ensue in ED (Lee et al., 2011; Joo et al., 2020). The keystone pathogens are key contributors for the host immune response subversion. For example, *P. gingivalis*, the keystone bacterium, possess virulence factors that can inactivate critical elements of the host response and enhance the proliferation and differentiation of Th-17 cells (Stein, 2015). However, further molecular mechanism exploration is still imperative and is promising for host homeostasis restoration that promote the resolution of inflammation.

Recently, new evidence has suggested that periodontitis can affect systemic health through the oral-intestinal axis, which might be a novel pathway independent of blood circulation (Bao et al., 2022). Whether periodontitis affects vascular endothelial health through this pathway is still unknown. However, there is a study that might be instructive. It detected the trimethylamine-N-oxide (TMAO), a harmful intestinal microbiota-dependent metabolite in periodontitis (Jalandra et al., 2021). In this research, elevated TMAO was presented in patients with stage III-IV periodontitis, and its concentration has been correlated with reduced circulating endothelial progenitor cells (EPCs) and FMD levels (Zhou et al., 2022). These data provide a novel perspective on the possibility of periodontitis affecting endothelial function through the oral-intestinal axis, which deserves deep exploration.

## Summary

**Figure 1:**
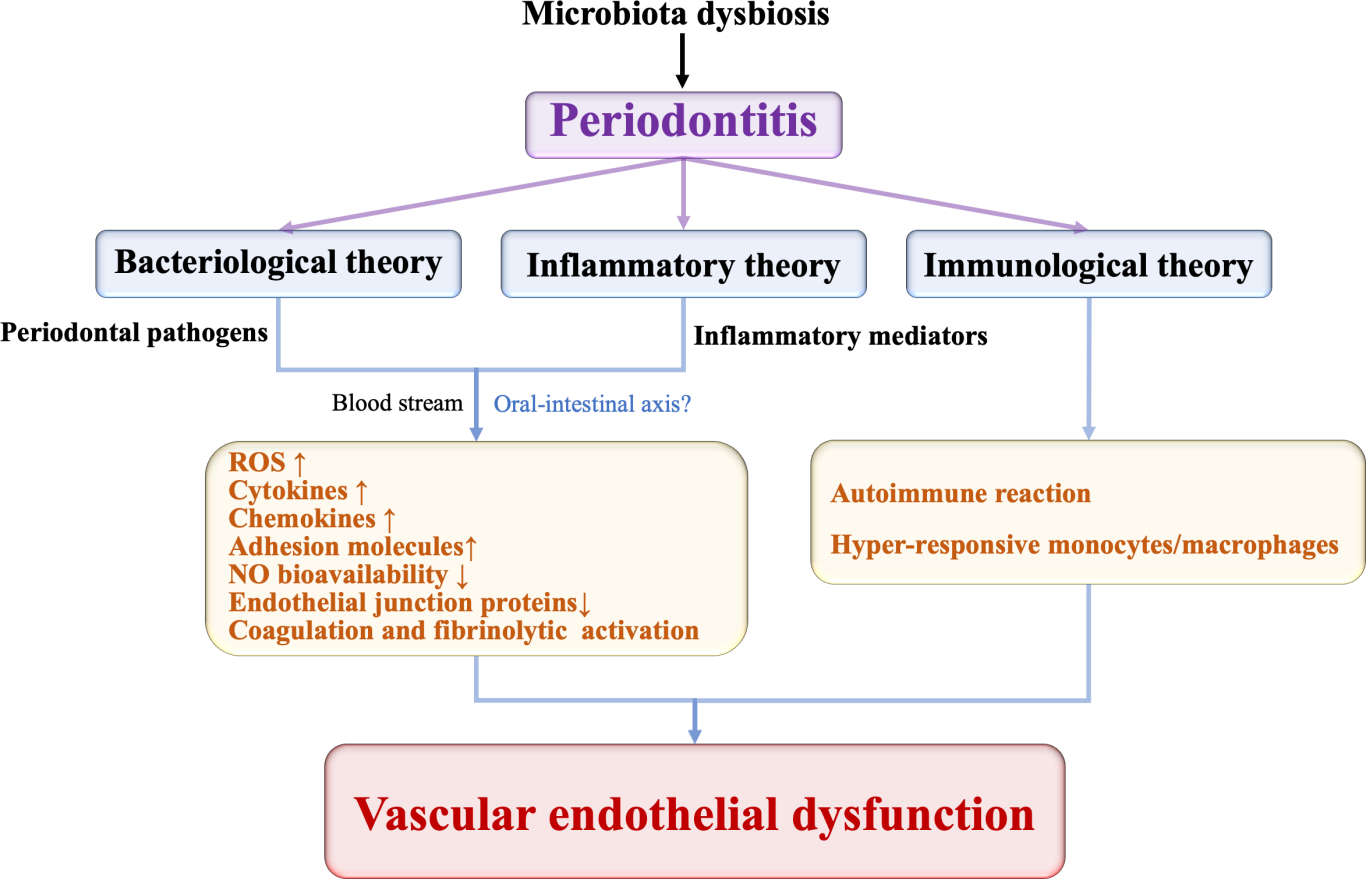
The potential pathways periodontitis induces vascular endothelial dysfunction.

This review summarizes current insights into the implication of periodontitis in ED (**Figure 1**). Current evidences suggest that periodontitis is highly associated with ED, which is of considerable importance for the risk and prognosis of cardiovascular diseases. Most of the epidemiological studies and clinical evidences have shown periodontal treatment as an effective measure for ED improvement. But more evidences are necessary for the impact of periodontal therapy on endothelial function in subjects with less widespread and severe periodontitis or with complex systemic conditions. Furthermore, the biological plausibility of periodontitis impact on vascular endothelium is being widely explored and gradually revealed. But much more researches are needed to elaborate the direct causal relationship between them.

To sum up, we still strongly recommend more collaboration between stomatologists and cardiologists in clinical work. Our stomatologists should pay more attention to the systemic health of patients and recommend them to visit cardiologists when necessary. At the same time, it is advocated that our general physicians attach importance to the oral health of patients and suggest them visit their stomatologists for periodontitis screening. If they are diagnosed with periodontitis, periodontal therapy is needed to improve their vascular endothelial function, thereby reducing the risk for cardiovascular disease events.

## Author contributions

QL wrote and edited this paper. XO and JL reviewed and edited the manuscript. All authors contributed to the article and approved the submitted version.

## Funding

This present work was supported by the National Natural Science Foundation of China (grant no. 82170957, 81870772), and by the Foundation of Beijing Tongren Hospital, Capital Medical University (grant no. 2021-YJJ-ZZL-047).

## Conflict of interest

The authors declare that the research was conducted in the absence of any commercial or financial relationships that could be construed as a potential conflict of interest.

The handling editor ZW declared a shared parent affiliation with the authors QL and JL at the time of review.

## Publisher’s note

All claims expressed in this article are solely those of the authors and do not necessarily represent those of their affiliated organizations, or those of the publisher, the editors and the reviewers. Any product that may be evaluated in this article, or claim that may be made by its manufacturer, is not guaranteed or endorsed by the publisher.
